# High coffee consumption and different brewing methods in relation to postmenopausal endometrial cancer risk in the Norwegian Women and Cancer Study: a population-based prospective study

**DOI:** 10.1186/1472-6874-14-48

**Published:** 2014-03-25

**Authors:** Oxana Gavrilyuk, Tonje Braaten, Guri Skeie, Elisabete Weiderpass, Vanessa Dumeaux, Eiliv Lund

**Affiliations:** 1 Department of Community Medicine, The Faculty of Health Sciences, The Arctic University of Norway, 9037 Tromsø, Norway; 2 Department of Medical Epidemiology and Biostatistics, Karolinska Institutet, Stockholm, Sweden; 3 Cancer Registry of Norway, Oslo, Norway; 4 Folkhälsan Research Centre, Samfundet Folkhälsan, Helsinki, Finland; 5 Department of Oncology, McGill University, Montreal, Canada

**Keywords:** Coffee, Brewing method, Endometrial cancer risk, Norway, High coffee consumption, Prospective cohort study

## Abstract

**Background:**

Coffee and its compounds have been proposed to inhibit endometrial carcinogenesis. Studies in the Norwegian population can be especially interesting due to the high coffee consumption and increasing incidence of endometrial cancer in the country.

**Methods:**

A total of 97 926 postmenopausal Norwegian women from the population-based prospective Norwegian Women and Cancer (NOWAC) Study, were included in the present analysis. We evaluated the general association between total coffee consumption and endometrial cancer risk as well as the possible impact of brewing method. Multivariate Cox regression analysis was used to estimate risks, and heterogeneity tests were performed to compare brewing methods.

**Results:**

During an average of 10.9 years of follow-up, 462 incident endometrial cancer cases were identified. After multivariate adjustment, significant risk reduction was found among participants who drank ≥8 cups/day of coffee with a hazard ratio of 0.52 (95% confidence interval, CI 0.34-0.79). However, we did not observe a significant dose-response relationship. No significant heterogeneity in risk was found when comparing filtered and boiled coffee brewing methods. A reduction in endometrial cancer risk was observed in subgroup analyses among participants who drank ≥8 cups/day and had a body mass index ≥25 kg/m^2^, and in current smokers.

**Conclusions:**

These data suggest that in this population with high coffee consumption, endometrial cancer risk decreases in women consuming ≥8 cups/day, independent of brewing method.

## Background

Endometrial cancer comprises about 4% of all cancers in women globally and occurs predominantly after menopause [1]. Some of the highest incidence rates worldwide are found in European populations [2], and a consistent increase in incidence has been observed in the Nordic countries [3]. Although the exact cause of this increase is unknown, use of postmenopausal estrogen therapy and exposure to high levels of obesity-related hormones (i.e. estrogen and insulin) may be contributing factors [4,5]. Recently, a growing number of studies have highlighted that coffee, one of the most consumed beverages in the world, may favorably alter hormone levels [6-9] and consequently lower endometrial cancer risk [10,11]. Coffee might also decrease endometrial cancer risk due to its antioxidant properties [12,13] and capability to prevent DNA damage [14]. Interestingly, coffee contains numerous active substances, such as caffeine, diterpenes, chlorogenic acids and phytoestrogens. Concentrations of caffeine and other coffee compounds vary by coffee type (e.g. caffeinated or decaffeinated) and might also be modified by different preparations/brewing methods (e.g. boiled or filtered) [15,16]. Although traditions concerning coffee types and brewing methods, and levels of coffee consumption may vary across populations, relatively few countries have investigated the association between specific coffee consumption and endometrial cancer risk.

Two recent prospective studies showed inconsistent results concerning the association between coffee type and endometrial cancer risk. While one study [17] found a significant decrease in risk associated with only caffeinated coffee in obese women, another [18] concluded that both caffeinated and decaffeinated coffee may reduce endometrial cancer risk. Overall, it is unclear whether caffeine is the compound causally associated with endometrial cancer risk reduction. Moreover, to the best of our knowledge, only one Swedish prospective study has investigated the effect of boiled and filtered coffee on endometrial cancer risk [19]. Although chemical analyses in several reports showed that filtration leads to an almost complete removal of diterpenes, the most promising chemopreventive compounds in coffee [16], the comparison of filtered and boiled coffee in the Swedish study did not reveal any differences between these brewing methods and endometrial cancer risk. Of note, results from the Swedish study were inconsistent with previous studies and found no significant effect of coffee consumption on endometrial cancer risk. Thus, it remains unclear what role, if any, different brewing methods or coffee types play when it comes to endometrial cancer risk.

Norway has the second largest coffee consumption per capita after Finland, including a long tradition of using different brewing methods [20,21]. Therefore our large prospective study based on a representative sample of the general Norwegian female population offers a unique opportunity to investigate the effect of high coffee consumption covering different brewing methods, including boiled and filtered, on the incidence of postmenopausal endometrial cancer, which is steadily increasing in Norway (about 700 new cases registered annually) [22].

## Methods

### The NOWAC cohort

Using the unique 11-digit personal identity number assigned to all people legally residing in Norway (citizens, those with temporary work permission, refugees, etc.), a random sample of women aged 30–70 years was chosen from the Central Population Registry of Norway and sent an invitation to participate in the Norwegian Women and Cancer (NOWAC) Study [23,24]. Linkage was then performed with the Central Population Registry to obtain postal addresses and vital status (alive, emigrated or deceased) of women. Linkage with the Cancer Registry of Norway was also performed to determine cancer incidence until December 31, 2009. The NOWAC Study is a large population-based cohort study, aim of which is to prospectively examine the associations between different lifestyle factors and the risk of various diseases in a representative sample of the general Norwegian female population [24]. Between 1991 and 2010, a total of 172 000 women were enrolled in the NOWAC Study. As part of their participation they completed a self-administered questionnaire on lifestyle, health and diet.

### Study sample

The present analysis used information taken from baseline questionnaires collected from NOWAC participants enrolled in 1991–1997 and 2003–2007 (129 854 women out of 239 388 invited, response rate 54.2%). Of these 129 854 women, we selected those who were either postmenopausal at start of follow-up, or who became postmenopausal during the course of follow-up. We excluded 2938 women with prevalent cancer at enrollment, 543 women who died before reaching menopause, 38 women with missing follow-up information on vital status and migration status, 6544 women who had hysterectomy at enrollment, 17 women who developed incident uterine sarcoma during follow-up, 7085 women with missing information on covariates, and 3090 premenopausal women (premenopausal at enrollment and throughout follow-up). We also excluded 11 673 women with missing information on coffee consumption. The final study sample included in the analyses consisted of 97 926 postmenopausal women at start of follow-up. All women provided informed consent, and the study was approved by The Regional Committee for Medical Research Ethics and the Norwegian Data Inspectorate.

### Dietary assessment

Year of enrollment determined the version of the questionnaire that participants completed, therefore women were asked about either their total coffee consumption (total coffee version of the questionnaire), or their consumption of filtered, boiled, and instant coffee separately (brewing method version of the questionnaire). The categories of coffee consumption were also different in two versions of the questionnaire. In the total coffee consumption version, women were asked to choose from one of the following responses: 6–10 cups/day, 4–5 cups/day, 2–3 cups/day, 1 cup/day, 5–6 cups/week, 2–4 cups/week, 1 cup/week, 1–3 cups/month, and almost never. However, in the brewing method version, women could also choose from the following responses: ≥8 cups/day, 6–7 cups/day, 4–5 cups/day, 2–3 cups/day, 1 cup/day, 1–6 cups/week, and almost never. But each woman had only one set of alternatives. Therefore, in our analysis we had to create a common version of frequencies for both the total coffee version and the brewing method version of the questionnaire. Therefore we collapsed the categories of lowest consumption from both versions of the questionnaire (1 cup/day, 5–6 cups/week, 2–4 cups/week, 1–6 cups/week, 1 cup/week, 1–3 cups /month and almost never) into a single category (≤1 cup/day), which was used as the reference in all analyses. The final categories used in the analysis were: ≤1 cup/day (reference category), 2–3 cups/day, 4–7 cups/day and ≥8 cups/day (heavy consumers). Participants who responded 6–10 cups/day in the total coffee questionnaire were categorized as ≥8 cups/day in the final categories. About 13% of the study population had missing values on brewing methods.

The size of a cup of coffee was not determined in the questionnaire, but we chose to use 2.1 dl (7.1 oz) as the standard cup size, based on a 24-hour recall investigation in the NOWAC cohort (data not shown). The NOWAC Study Food Frequency Questionnaire (FFQ) has been thoroughly validated by 24-h recalls [25] and a test-retest study [26], which showed good reproducibility and validity of information on coffee consumption (Spearman’s rank correlation coefficient r = 0.82, 95% confidence interval, CI 0.77-0.86; calibration coefficient 1.17, 95% CI 1.06-1.28).

### Assessment of lifestyle factors

Information on age at inclusion, age at menarche, age at first birth, education level, duration of oral contraceptive use, hormone replacement therapy use (current, former, never), smoking status (current, former, never), physical activity, and current weight and height were collected from questionnaire at enrollment. Body mass index (BMI) was calculated as weight in kg divided by height in meters squared. Menopausal status was updated when information was available. Women who were premenopausal at enrollment, but whose menopausal status changed to postmenopausal during follow-up contributed to the study as from the date they became postmenopausal. Menopausal status was derived from the answers to questions on menstruation regularity in the questionnaires. If women reported that their menstruation was regular, they were considered premenopausal. Women were classified as postmenopausal if they reported that their menstruation had stopped at the time of enrollment or during the follow-up. In case of uncertain menstruation regularity (irregular, hysterectomy, hormone replacement therapy use or otherwise insufficient information), women were defined as postmenopausal if they were at least 53 years old at the time of enrollment, or once they turned 53 during follow-up. This cut-off point has been used in previous NOWAC reports [23], based on the definition employed in The Million Women Study [27].

### Ascertainment of endometrial cancer cases

Information on cancer incidence among NOWAC Study participants was obtained by yearly linkage to the Norwegian Cancer Registry based on the unique 11-digit person identification number assigned to each Norwegian legal resident and citizen. By the end of the follow-up period, 462 incident endometrial cancer cases were recorded in our study sample.

### Statistical analysis

Follow-up of participants started at age of enrollment in the NOWAC Study (1991-1997, 2003–2007) or at age at menopause (for those women who were premenopausal at enrollment). Women were censored at the date of diagnosis of endometrial cancer, date of death or immigration, date of report of any other cancer, or the end of follow-up (31 December 2010). Cox proportional hazard regression models were used to estimate the hazard ratios (HRs) of developing endometrial cancer with 95% CIs in each category of coffee consumption compared with consumers in the lowest category of consumption (≤1 cup/day). We were unable to use coffee abstainers (non-consumers) as a reference group as we had too few endometrial cancer cases in the groups of only boiled and only instant coffee drinkers (Table 1). The variable total coffee consumption included responses to total coffee consumption (from total coffee version of the questionnaire) or a combination of responses to boiled, filtered and instant coffee consumption (from the brewing method version of the questionnaire). As there were very few heavy consumers of instant coffee (n = 2), we did not investigate the effect of instant coffee consumption when estimating risk stratified by brewing method. However, instant coffee consumption was included in total coffee consumption. A test of the proportional hazard assumption was checked graphically and by assessment of Schoenfeld residuals for all relevant variables.

**Table 1:** Distribution of NOWAC postmenopausal participants according to consumption of total, filtered, boiled and instant coffee

	**Total coffee, %**^**a**^	**Boiled coffee**		**Filtered coffee**		**Instant coffee**	
		**Combined with other brewing methods, %**^**b**^	**Only boiled coffee, %**	**Combined with other brewing methods, %**	**Only filtered coffee, %**	**Combined with other brewing methods, %**	**Only instant coffee, %**
No of participants	n = 97 926	n = 93 858	n = 32 049	n = 93 858	n = 60 025	n = 93 858	n = 22 501
≤ 1 cups per day	16.1	80.0	54.4	40.1	25.6	91.7	77.5
2-3 cups per day	32.9	7.9	16.1	25.9	30.4	5.4	13.3
4-7 cups per day	37.8	8.4	20.2	25.9	33.4	2.4	7.5
≥ 8 cups per day	13.2	3.7	9.3	8.1	10.6	0.5	1.7
No of cases	n = 462	n = 452	n = 174	n = 452	n = 322	n = 452	n = 112
≤ 1 cups per day	17.7	77.9	51.2	44.2	27.6	92.7	79.5
2-3 cups per day	37.1	10.4	21.8	27.0	34.2	4.6	10.7
4-7 cups per day	38.3	10.2	23.0	25.0	32.9	2.3	8.0
≥ 8 cups per day	6.9	1.5	4.0	3.8	5.3	0.4	1.8

^a^Total coffee is combination of responses on total coffee consumption in the total coffee version of the questionnaire and filtered, boiled and instant coffee consumption in the brewing methods version of the questionnaire. ^b^Combined with other types: those women who positively responded to question about “total coffee consumption” or/and other brewing method (filtered, boiled, instant).

To test for trends, we looked at the percentage points of risk reduction conferred by each additional cup of coffee consumed when consumption was coded as a continuous variable or we assigned a median value to each category of coffee consumption when coded as a categorical variable. Multivariate analyses were carried out to control for the potential confounding effects of age at first birth, parity, age at menopause, oral contraceptive use, hormone replacement therapy use, smoking status, and BMI. We assessed the heterogeneity in effects between different brewing methods by Wald tests. Because of the proposed modifying role of BMI and smoking status, we performed subgroup analyses estimating the risk conferred by total coffee consumption on endometrial cancer stratified by these factors although we did not find any significant interaction between total coffee consumption and smoking (p = 0.72) and total coffee consumption and BMI (p = 0.66). We also performed analyses in which boiled and filtered coffee were adjusted for each other, using coffee consumption as both a categorical and a continuous variable. All analyses were conducted using SAS for Windows (version 9.2; SAS Institute Inc., Cary, North Carolina, USA). All tests were two-sided with statistical significance set at p < 0.05.

## Results

### Distribution of NOWAC participants and baseline characteristics

Participants who answered on brewing method version of the questionnaire were divided into two subgroups: those who consumed coffee prepared by only one brewing method (boiled, filtered, or instant) and those who used a mixture of brewing methods.

Among all coffee consumers, women with total coffee consumption of 2–3 cups/day and 4–7 cups/day represented the largest groups (32.9% and 37.8%, respectively). Although women drinking only filtered coffee also mostly reported to drink between 2–7 cups/day women drinking only boiled coffee appear to consume less with 54.4% drinking ≤1 cup/day (Table 1). In the group that we classified as non-consumers, there was a substantial proportion of women who were actually instant coffee drinkers in very small quantities (91.7% of participants in the group of ≤1 cup/day).

Table 2 shows the distribution of known risk factors and main baseline characteristics of our study cohort according to total coffee consumption. Women with a higher total coffee consumption had lower age at enrollment, at menarche, at first birth and at menopause, were less often nulliparous, were more often current smokers, and were leaner than women with lower coffee consumption. This tendency was observed within all brewing methods. During an average of 10.9 years of follow-up, 462 cases of incident endometrial cancer were diagnosed in 97 926 women, contributing 1 071 943 person-years of observation. The mean age at diagnosis of endometrial cancer was 61.5 years (Table 2).

**Table 2:** Baseline characteristics of postmenopausal NOWAC Study participants according to total coffee consumption

**Characteristics**	**Average total coffee consumption, cups per day**			
	**≤ 1**	**2-3**	**4-7**	**≥ 8**
Number of women (%)	15795 (16.1)	32200 (32.9)	36992 (37.8)	12939 (13.2)
Number of EC cases (%)	82 (17.7)	171 (37.1)	177 (37.8)	32 (6.9)
Mean (sd)				
Age at enrollment, years	47.5 (8.64)	48,7 (8.73)	47.9 (8.31)	43.7 (6.82)
Age at menarche, years	13.4 (1.45)	13.4 (1.37)	13.3 (1.36)	13.2 (1.39)
Age at first birth, years	24.8 (4.53)	24.4 (4.39)	23.6 (4.17)	22.7 (3.99)
Age at menopause, years	48.1 (5.03)	48.7 (8.74)	48.3 (4.66)	46.9 (4.95)
Duration of OC use, years	2.9 (4.56)	2.6 (4.29)	2.5 (4.12)	2.6 (4.01)
Number of children, %				
0	11.4	9.5	7.9	7.5
1-2	55.4	53.9	52.6	53.5
≥ 3	33.2	36.6	39.5	39.0
Years of total education, %				
< 9	16.6	21.2	28.9	36.7
10-12	30.2	33.3	36.3	38.3
13-16	32.5	29.9	24.3	19.3
> 17	20.8	15.6	10.5	5.9
Smoking status, %				
Current	17.7	21.8	39.3	61.8
Former	31.1	35.6	31.9	24.6
Never	51.2	42.6	28.8	13.7
BMI, %				
< 20	10.1	8.1	7.9	10.9
20-24.9	56.8	58.1	56.7	57.6
25-29.9	24.3	26.7	28.3	24.8
≥ 30	8.8	7.1	7.0	6.8
HRT use, %				
Never	79.7	79.9	83.3	91.1
Former	8.7	8.4	7.1	3.3
Current	11.6	11.7	9.7	5.6
Prevalence of diabetes, %	0.98	0.82	0.92	0.98

*Abbreviations*: *NOWAC* Norwegian Women and Cancer, *EC* Endometrial cancer, *SD* Standard deviation, *OC* Oral contraceptives, *BMI* Body mass index, *HRT* Hormone replacement therapy.

N = 97 926.

### Endometrial cancer risk

A statistically significant inverse association between total coffee consumption and endometrial cancer risk was observed among heavy consumers (age-adjusted HR 0.44, 95% CI 0.29-0.67) (Table 3). This association was slightly attenuated after multivariate adjustment (HR 0.52, 95% CI 0.34-0.79).

**Table 3:** HRs and 95% CIs of endometrial cancer related to total, filtered and boiled coffee among 97 926 postmenopausal NOWAC Study participants

**No of cups**	**Total coffee**^**a**^	**Boiled coffee**		**Filtered coffee**	
		**Combined with other types**^**b**^	**Only boiled coffee**	**Combined with other types**	**Only filtered coffee**
Age-adjusted HR, CI 95%^c^					
≤ 1 cups/day	1.0 (ref)	1.0 (ref)	1.0 (ref)	1.0 (ref)	1.0 (ref)
2-3 cups/day	0.87 (0.67-1.13)	1.12 (0.83-1.53)	1.03 (0.70-1.51)	0.98 (0.79-1.23)	0.93 (0.70-1.23)
4-7 cups/day	0.76 (0.59-0.99)	1.06 (0.78-1.44)	0.87 (0.59-1.26)	0.89 (0.71-1.13)	0.79 (0.60-1.06)
≥ 8 cups/day	0.44 (0.29-0.67)	0.43 (0.2-0.91)	0.42 (0.19-0.91)	0.48 (0.29-0.79)	0.45 (0.26-0.75)
p for trend	< 0.0001	0.21	0.02	0.002	0.03
Multivariate-adjusted HR, CI 95%^d^					
≤ 1 cups/day	1.0 (ref)	1.0 (ref)	1.0 (ref)	1.0 (ref)	1.0 (ref)
2-3 cups/day	0.91 (0.70-1.19)	1.08 (0.79-1.46)	1.04 (0.71-1.54)	0.99 (0.79-1.24)	0.99 (0.75-1.31)
4-7 cups/day	0.84 (0.65-1.1)	1.10 (0.81-1.50)	0.92 (0.62-1.36)	0.95(0.76-1.20)	0.91 (0.68-1.21)
≥ 8 cups/day	0.52 (0.34-0.79)	0.48 (0.23-1.03)	0.45 (0.21-1.01)	0.55 (0.33-0.91)	0.55 (0.32-0.94)
p for trend	0.003	0.36	0.07	0.12	0.07

^a^Total coffee is combination of responses on total coffee consumption in the total coffee version of the questionnaire and filtered, boiled and instant coffee consumption in the brewing methods version of the questionnaire. ^b^Combined with other types: those women who positively responded to question about “total coffee consumption” or/and other brewing method (filtered, boiled, instant). ^c^Basic model that was adjusted for age. ^d^Multivariate model that was adjusted for parity, smoking status, BMI, duration of OC and HRT use. *Abbreviations*: *HR* Hazard ratio, *CI* Confidence interval, *NOWAC* Norwegian Women and Cancer, *BMI* Body mass index, *OC* Oral contraceptives, *HRT* Hormone replacement therapy.

When analyzing different brewing methods, there was an inverse association among heavy consumers of filtered coffee only and heavy consumers of filtered coffee combined with other coffee brewing methods (multivariate-adjusted HR 0.55, 95% CI 0.32-0.94 and HR 0.55, 95% CI 0.33-0.91 respectively). However, when frequency of filtered coffee consumption was analyzed as a continuous variable, no significant dose-response relationship was observed for filtered coffee alone (p for trend 0.07), or for filtered coffee combined with other brewing methods (p for trend 0.12). In boiled coffee drinkers we observed a significant inverse association only among heavy consumers (age-adjusted HR 0.42, 95% CI 0.19-0.91), but this inverse association was borderline significant after multivariate adjustment (HR 0.45, 95% CI 0.21-1.01). We did not observe any significant dose-response relationship when boiled coffee consumption alone, or boiled coffee combined with other brewing methods were included as continuous variables (p for trend 0.07 and 0.36 respectively).

Since BMI is a strong risk factor for endometrial cancer, and smoking has been reported to alter caffeine clearance, we examined additional effect modification by stratifying analyses by brewing method, BMI (<25 and ≥25, which is considered overweight) and smoking status (never, former, current). Total coffee consumption was significantly inversely associated with endometrial cancer only among overweight women who were heavy total coffee consumers or heavy consumers of filtered coffee (multivariate-adjusted HR 0.39, 95% CI 0.21-0.73 and HR 0.46, 95% CI 0.22-0.96, respectively) (Table 4). There was a significant reduction in endometrial cancer risk among current smokers who were heavy total coffee consumers (multivariate-adjusted HR 0.36, 95% CI 0.17-0.79). However, the association between smoking and endometrial cancer risk in relation to different brewing methods did not reach statistical significance, which could be due to limited statistical power. Stratified analysis by history of diabetes mellitus, physical activity and hormone replacement therapy use was hampered by small numbers. Nevertheless, when analyses were restricted to non-diabetic women, results were similar to those from the entire study sample. To eliminate the potential effects of early undiagnosed endometrial cancer, we repeated our analysis, excluding endometrial cancer diagnosed during the first year of follow-up. Results from this analysis did not differ from those for the entire cohort. Adjusting one coffee brewing method by another method did not affect any of the risk analyses (Additional file 1: Table S1). No significant differences between boiled coffee and filtered coffee were found (Additional file 2: Table S2).

**Table 4:** HRs and 95% CIs of total, boiled and filtered coffee consumption stratified by BMI and smoking status in 97 926 postmenopausal NOWAC Study participants

	**No of cups**	**Age-adjusted HR, CI 95%**^**a**^			**Multivariate-adjusted HR, CI 95%**^**b**^		
		**Total coffee**	**Boiled coffee**	**Filtered coffee**	**Total coffee**	**Boiled coffee**	**Filtered coffee**
BMI < 25	≤ 1	1.0 (ref)	1.0 (ref)	1.0 (ref)	1.0 (ref)	1.0 (ref)	1.0 (ref)
	2-3	0.93 (0.63-1.38)	0.88 (0.53-1.47)	1.29 (0.93-1.79)	0.96 (0.65-1.42)	0.87 (0.52-1.46)	1.25 (0.90-1.74)
	4-7	0.78 (0.53-1.16)	0.88 (0.53-1.44)	1.05 (0.75-1.49)	0.86 (0.58-1.29)	0.97 (0.59-1.60)	1.11 (0.78-1.57)
	≥ 8	0.54 (0.31-0.96)	0.50 (0.19-1.36)	0.57 (0.29-1.14)	0.65 (0.36-1.17)	0.59 (0.22-1.63)	0.66 (0.33-1.32)
p for trend		0.009	0.13	0.39	0.008	0.31	0.77
BMI ≥ 25	≤ 1	1.0 (ref)	1.0 (ref)	1.0 (ref)	1.0 (ref)	1.0 (ref)	1.0 (ref)
	2-3	0.82 (0.57-1.17)	1.26 (0.85-1.85)	0.79 (0.58-1.09)	0.83 (0.58-1.19)	1.22 (0.83-1.80)	0.78 (0.57-1.08)
	4-7	0.72 (0.51-1.02)	1.13 (0.77-1.67)	0.72 (0.51-1.02)	0.78 (0.55-1.12)	1.21 (0.82-1.79)	0.83 (0.60-1.13)
	≥ 8	0.33 (0.18-0.61)	0.33 (0.10-1.02)	0.40 (0.19-0.83)	0.39 (0.21-0.73)	0.39 (0.12-1.23)	0.46 (0.22-0.96)
p for trend		0.0005	0.44	0.01	0.009	0.79	0.05
Never smokers	≤ 1	1.0 (ref)	1.0 (ref)	1.0 (ref)	1.0 (ref)	1.0 (ref)	1.0 (ref)
	2-3	1.01 (0.7-1.44)	1.09 (0.72-1.67)	1.15 (0.85-1.57)	1.05 (0.74-1.51)	1.07 (0.70-1.62)	1.19 (0.87-1.62)
	4-7	0.97 (0.67-1.41)	0.95 (0.57-1.58)	0.08 (0.76-1.53)	0.97 (0.67-1.42)	0.91 (0.55-1.53)	1.08 (0.76-1.53)
	≥ 8	0.54 (0.23-1.26)	0.36 (0.05-2.52)	0.67 (0.25-1.83)	0.49 (0.21-1.16)	0.33 (0.05-2.33)	0.63 (0.23-1.72)
p for trend		0.41	0.59	0.99	0.26	0.44	0.94
Former smokers	≤ 1	1.0 (ref)	1.0 (ref)	1.0 (ref)	1.0 (ref)	1.0 (ref)	1.0 (ref)
	2-3	0.75 (0.47-1.19)	1.05 (0.59-1.86)	0.69 (0.45-1.05)	0.76 (0.47-1.21)	1.04 (0.58-1.85)	0.70 (0.46-1.07)
	4-7	0.76 (0.48-1.21)	1.79 (1.12-2.85)	0.79 (0.52-1.18)	0.74 (0.46-1.18)	1.69 (1.06-2.72)	0.77 (0.51-1.16)
	≥ 8	0.59 (0.29-1.20)	0.25 (0.03-1.76)	0.66 (0.30-1.45)	0.57 (0.28-1.15)	0.23 (0.03-1.67)	0.64 (0.29-1.42)
p for trend		0.09	0.67	0.19	0.06	0.83	0.18
Current smokers	≤ 1	1.0 (ref)	1.0 (ref)	1.0 (ref)	1.0 (ref)	1.0 (ref)	1.0 (ref)
	2-3	0.73 (0.37-1.45)	1.16 (0.56-2.41)	1.04 (0.60-1.78)	0.76 (0.38-1.52)	1.16 (0.56-2.42)	1.04 (0.60-1.79)
	4-7	0.61 (0.32-1.15)	0.76 (0.39-1.48)	0.93 (0.58-1.50)	0.64 (0.34-1.21)	0.77 (0.39-1.50)	0.96 (0.59-1.55)
	≥ 8	0.36 (0.17-0.79)	0.69 (0.28-1.73)	0.40 (0.17-0.95)	0.37 (0.17-0.81)	0.69 (0.28-1.73)	0.40 (0.17-0.95)
p for trend		0.009	0.30	0.12	0.01	0.30	0.13

^a^Basic model that was adjusted for age. ^b^Multivariate model that was adjusted for parity, smoking status, BMI, duration of OC and HRT use. *Abbreviations*: *HR* Hazard ratio, *CI* Confidence interval, *BMI* Body mass index, *NOWAC* Norwegian Women and Cancer, *OC* Oral contraceptives, *HRT* Hormone replacement therapy.

## Discussion

In this large population-based prospective study of postmenopausal Norwegian women, total coffee consumption was inversely associated with endometrial cancer risk, whereas there were no significant differences in this association by considered coffee brewing methods. The inverse association was stronger among overweight women, current smokers and heavy consumers. The current results show an overall association between total coffee consumption and endometrial cancer risk, which corroborates the findings of recent prospective studies [10,28-30] and supports the hypothesis that coffee and its compounds may play an important role in the chemoprevention of endometrial carcinogenesis.

There are several strengths in our study. The NOWAC cohort is well-characterized with a large number of participants who were randomly selected from the general population, giving our study enough statistical power to detect small differences in the studied subgroups. Lifestyle and dietary data were collected prior to endometrial cancer diagnosis. This minimizes the risk of recall bias, selection bias and reverse causation. In addition, we had a broad range of exposures in the cohort, and information on several coffee brewing methods. To our knowledge this is the largest cohort study to investigate the association between endometrial cancer risk and boiled/filtered coffee consumption. Finally, our cohort was based in a population with strong coffee consumption habits and low number of non-consumers.

Our study also has some limitations, such as the self-reporting of coffee consumption. Even though results from both the NOWAC Study [25] and other studies [31,32] have shown a satisfactory reproducibility and validity of information on coffee consumption, various biases may still arise when self-reported FFQs are used. However, this kind of misclassification would most probably weaken the studied association, and would not lead to significant bias. Other limitations are the lack of information on the newer types of coffee drinks that have been incorporated into the Norwegian diet in the last decade (e.g. cappuccino, mocha and latte), and the lack of information about decaffeinated coffee, which, however, was rare in Norway during the enrollment periods. Moreover, we lacked data on how coffee beans were roasted, which can also influence the level and properties of some coffee compounds [16,33]. Finally, for a large part of the cohort there is no detailed information on consumption of tea, cola-type drinks, and chocolate, which contain some of the same possible bioactive compounds as coffee, and therefore these variables could not be adjusted for the present analysis.

Over the past two decades several studies have evaluated the association between coffee consumption and endometrial cancer risk. Only one prospective study from Sweden has investigated the association between two coffee brewing methods and endometrial cancer risk [19] and did not find any significant decrease in endometrial cancer risk associated with coffee consumption. Our results support the main hypothesis of recent prospective studies [10,28], which suggested a significant inverse association with total coffee consumption, in particular among overweight women. However, these studies reported a protective effect with moderate coffee consumption (3–4 cups/day) [10,28], whereas we only observed this effect at high consumption levels (≥8 cups/day). This discrepancy might be explained by the fact that Scandinavians used to drink far more coffee than people in other countries [34,35]. Recent investigations from the National Coffee Information Organization showed that in the period 1982–2009, which includes the enrolment period of the NOWAC Study, 90% of Norwegians aged over 40 years had an average coffee consumption of 4–5 cups/day [21]. It is possible that such a long tradition of heavy consumption changed mechanisms involved in the association between coffee consumption and cancer risk. This hypothesis however, needs further exploration. We cannot exclude the possibility that coffee consumption reported at enrollment decreases during follow-up for most participants.

There are several potential theories by which coffee may reduce endometrial cancer risk. The hormonal theory asserts that the metabolism of caffeine and other coffee compounds interact mainly with the metabolism of estrogens. The oxidative metabolism of both caffeine and estrogens are regulated by the same family of cytochrome enzymes, and there is an interaction between them [36-39]. In addition, the hormonal mechanism focuses on the ability of coffee to regulate several hormone levels, the altered stimulation of which may lead to hyperinsulinemia and, therefore, increased proliferation of the endometrial stromal cells [40,41]. Thus, coffee increases the level of adiponectin, the deficiency of which leads to hyperinsulinemia [42], increases the level of free estrogens by decreasing the level of sex hormone-binding globulin (SHBG), and increases the level of circulating free insulin-like growth factor-1 by decreasing the levels of insulin-like growth factor-binding protein [9]. Another theory suggests that coffee compounds block the initiation phase of the carcinogenic process, decrease DNA damage and protect cells against reactive oxygen species by inducing the production of detoxifying enzymes through several mechanisms [43,44].

The question about which bioactive compounds in coffee have a higher antioxidant capacity remains open. Exposure to these coffee components is highly dependent on different conditions such as choice of beans and phase of administration, and is most likely also dependent on brewing method [16,33]. For a long time in Norway, boiled coffee, which is prepared by boiling water and adding coarse grounds in a pot, was the traditional brewing method. However, since boiled coffee may increase cholesterol levels [15], this brewing method was, to a large extent, replaced by filtered coffee starting in the 1990s. Based upon the market information from the National Coffee Information Organization [21], Norway has been mostly importing coffee Arabica, which is known to contain a higher percentage of diterpenes, but a lower concentration of caffeine in comparison to coffee Robusta [16]. In some studies it was highlighted that use of different methods and conditions can change the chemical composition of coffee and therefore open opportunities to modify and design various coffee brews with desired beneficial health effects [15]. Despite of this, we did not observe any significant favorable differences in the brewing methods we considered in our report. On another note, the negative effects of coffee drinking on health cannot be ignored. Indeed, high amounts of boiled coffee may increase cholesterol levels [45], although the beneficial effects of coffee on health might be expected at consumption levels that do not entail any increase in blood cholesterol [46].

In subgroup analyses, our data suggested that mechanisms involving interactions with BMI and smoking may be relevant in the association between coffee consumption and endometrial cancer risk. Indeed, overweight women with high coffee consumption have already been reported to have stronger protection against endometrial cancer in comparison to lean women [10,28]. This could be explained by the fact that obese women already have lower levels of SHBG, resulting in higher levels of bioavailable estrogen, hypoadiponectinemia, insulin resistance, hyperinsuliemia, and high levels of oxidative stress [47], all of which have been reported to improve in coffee drinkers [9,42]. It is also of interest that in our study there was a strong significant inverse association between coffee drinking and endometrial cancer risk among women who were current smokers. These results are consistent with the results from a recently published study [10], but the underlying mechanisms are not fully understood. It is known that caffeine, nicotine and estrogens are metabolized by the same family of cytochrome P450 enzymes [48,49]. As the half-life of caffeine is much lower in smokers than non-smokers, caffeine intake is usually higher in smokers. It was also reported that smoking induces the 2-hydroxylation pathway of estradiol metabolism, leading to decreased bioavailability at estrogen target tissues. Thus, it was suggested that caffeine in combination with nicotine enhances the clearance of estradiol by increasing CYP1A2 activity, and, hence, the combination of coffee drinking and smoking might offer more pronounced protection against hormone-dependent tumors [50]. In addition to high BMI and smoking, some studies on the association between endometrial cancer and coffee consumption have reported a stronger association among never users, or at least former users, of hormone replacement therapy [10,28]. We did not observe any significant differences in our subgroup analyses for ever and never users of hormone replacement therapy but our analysis was hampered by small numbers.

## Conclusions

In conclusion, the findings of our study are in line with previous reports indicating that total coffee consumption may decrease endometrial cancer risk. In addition, we found no significant heterogeneity in risk when comparing different brewing methods (filtered and boiled coffee). Because our population generally has a high coffee consumption at inclusion, the observed decreased endometrial cancer risk among women consuming ≥8 cups/day should be considered with caution. Thus, these findings need confirmation in other populations with consistent heavy coffee consumption.

## Abbreviations

BMI: Body mass index; CI: Confidence interval; HR: Hazard ratio; NOWAC: Norwegian Women and Cancer; SBHG: Sex hormone-binding globulin.

## Competing interests

The authors declare that they have no competing interests.

## Authors’ contributions

OG carried out the interpretation of the data and drafted the manuscript. TB carried out the statistical analysis. EL developed the research plan, directed the analysis and contributed with critical revision of the manuscript. He is the principal investigator and designed the NOWAC Study. VD contributed with the interpretation of the data and helped to draft the manuscript. GS contributed with the planning of statistical analysis, consulted in nutrition questions and helped in revision of the manuscript. EW contributed with planning the statistical analysis, interpretation of the data, and revision of the manuscript. All authors read and approved the final manuscript.

## Pre-publication history

The pre-publication history for this paper can be accessed here:

http://www.biomedcentral.com/1472-6874/14/48/prepub

## Supplementary Material

Additional file 1 **Age-adjusted and multivariate-adjusted HRs and 95% CIs.** One coffee brewing method adjusted for other brewing methods. Click here for file

Additional file 2 **Test for heterogeneity.** Comparison between heavy boiled coffee drinkers only and heavy filtered coffee drinkers only. Click here for file
