# Supplementary material for: High coffee consumption and different brewing methods in relation to postmenopausal endometrial cancer risk in the Norwegian Women and Cancer Study: a population-based prospective study
Source: BMC Womens Health. 2014 Mar 25;14:48. doi: 10.1186/1472-6874-14-48 (PMC3986939; doi:10.1186/1472-6874-14-48)
Supplement: Additional file 1 — Age-adjusted and multivariate-adjusted HRs and 95% CIs. One coffee brewing method adjusted for other brewing methods. [file 1472-6874-14-48-S1.docx]

**Supplementary table 1 - Age-adjusted and multivariate-adjusted HRs and 95% CIs. One coffee brewing method adjusted for other brewing methods**

| Coffee groups compaired | Age-adjusted HR (CI 95%), p | Multivariate-adjusted HR (CI 95%), p |
| --- | --- | --- |
| Boiled coffee adjusted for filtered coffee continuous (86 100 women, 419 cases) | | |
| ≤ 1 cups per day of boiled coffee | 1.0 (ref) | 1.0 (ref) |
| 2-3 cups per day of boiled coffee | 0.92 (0.66-1.28), p = 0.61 | 0.95 (0.68-1.33), p = 0.76 |
| 4-7 cups per day of boiled coffee | 0.85 (0.61-1.28), p = 0.33 | 0.96 (0.69-1.36), p = 0.85 |
| ≥ 8 cups per day of boiled coffee | 0.35 (0.16-0.74), p = 0.006 | 0.43 (0.19-0.92), p = 0.03 |
| Number of cups per day of filtered coffee-continuous variable | 0.93 (0.88-0.97), p = 0.002 | 0.95 (0.91-0.99), p < 0.001 |
| Filtered coffee adjusted for boiled coffee continuous (86 100 women, 419 cases) | | |
| ≤ 1 cups per day of filtered coffee | 1.0 (ref) | 1.0 (ref) |
| 2-3 cups per day of filtered coffee | 0.83 (0.65-1.07), p = 0.15 | 0.88 (0.68-1.13), p = 0.31 |
| 4-7 cups per day of filtered coffee | 0.74 (0.57-0.97), p = 0.03 | 0.83 (0.64-1.09), p = 0.18 |
| ≥ 8 cups per day of filtered coffee | 0.39 (0.39-0.67), p = 0.0004 | 0.48 (0.29-0.81), p = 0.006 |
| Number of cups per day of boiled coffee-continuous variable | 0.93 (0.88-0.98), p = 0.009 | 0.95 (0.89-1.01), p = 0.09 |
| Filtered coffee continuous adjusted for boiled coffee continuous (86 100 women, 419 cases) | | |
| Numbers of cups per day of filtered coffee-continuous variable | 0.92 (0.88-0.96), p = 0.003 | 0.94 (0.89-0.98), p = 0.01 |
| Numbers of cups per day of boiled coffee-continuous variable | 0.92 (0.87-0.98), p = 0.005 | 0.95 (0.89-1.03), p = 0.06 |

Abbreviations: HR: hazard ratio; CI: confidence interval; NOWAC: Norwegian Women and Cancer.
