# Supplementary material for: High coffee consumption and different brewing methods in relation to postmenopausal endometrial cancer risk in the Norwegian Women and Cancer Study: a population-based prospective study
Source: BMC Womens Health. 2014 Mar 25;14:48. doi: 10.1186/1472-6874-14-48 (PMC3986939; doi:10.1186/1472-6874-14-48)
Supplement: Additional file 2 — Test for heterogeneity. Comparison between heavy boiled coffee drinkers only and heavy filtered coffee drinkers only. [file 1472-6874-14-48-S2.docx]

**Supplementary table 2 - Test for heterogeneity. Comparison between heavy boiled coffee drinkers only and heavy filtered coffee drinkers only**

|  | Age-adjusted HR, 95% CI | Multivariate-adjusted HR, 95% CI |
| --- | --- | --- |
| Heavy boiled coffee drinkers only | 0.41 (0.18-0.92), p = 0.03 | 0.50 (0.21-1.21), p = 0.12 |
| Heavy filtered coffee drinkers only | 0.40 (0.22-0.74), p = 0.003 | 0.49 (0.26-0.98), p = 0.04 |

Abbreviations: HR: hazard ratio; CI: confidence interval.
